# Ulcerated Radiodermatitis Induced after Fluoroscopically Guided Stent Implantation Angioplasty

**DOI:** 10.1155/2014/768624

**Published:** 2014-09-03

**Authors:** Maira Elizabeth Herz-Ruelas, Minerva Gómez-Flores, Joaquín Moxica-del Angel, Ivett Miranda-Maldonado, Ilse Marilú Gutiérrez-Villarreal, Guillermo Antonio Guerrero-González, Adriana Orelia Villarreal-Rodríguez

**Affiliations:** ^1^Dermatology Department, Hospital Universitario “Dr. José Eleuterio González,” Universidad Autónoma de Nuevo León, Monterrey, Mexico; ^2^Christus Mugerza Sur Hospital, Monterrey, Mexico; ^3^Pathology Department, Hospital Universitario “Dr. José Eleuterio González,” Universidad Autónoma de Nuevo León, Monterrey, Mexico; ^4^School of Medicine, Universidad Autónoma de Nuevo León, Monterrey, Mexico

## Abstract

Cases of radiation-induced skin injury after fluoroscopically guided procedures have been reported since 1996, though the majority of them have been published in Radiology and Cardiology literature, less frequently in Dermatology journals. Chronic radiation dermatitis induced by fluoroscopy can be difficult to diagnose; a high grade of suspicion is required. We report a case of an obese 46-year-old man with hypertension, dyslipidemia, and severe coronary artery disease. He developed a pruritic and painful atrophic ulcerated skin plaque over his left scapula, six months after fluoroscopically guided stent implantation angioplasty. The diagnosis of radiodermatitis was confirmed histologically. We report this case to emphasize the importance of recognizing fluoroscopy as a cause of radiation dermatitis. A good clinical follow-up at regular intervals is important after long and complicated procedures, since the most prevalent factor for injury is long exposure time.

## 1. Introduction

Cases of radiation-induced skin injury after fluoroscopically guided procedures have been reported since 1996; however, diagnosis and treatment of such lesions remain difficult [1]. Fluoroscopy-induced chronic radiation dermatitis often requires a high clinical suspicion to establish a correct diagnosis [2]. Ionizing radiation during interventional procedures is often underestimated. The risk of developing this reaction is directly related to the radiation dose, which depends on the type of procedure, technique, time of exposure, and the patient's body constitution [3]. The period between radiation exposure and manifestation of skin injuries varies, from 15 days up to months or years.

The incidence of radiodermatitis after percutaneous coronary interventions by X-ray fluoroscopic procedures is rising; case reports have been increasingly documented. The skin lesions encompass a wide spectrum, such as erythema, telangiectasias, atrophy, hyperpigmentation and hypopigmentation, necrosis, chronic ulceration, and squamous cell carcinoma [4].

Chronic radiation dermatitis induced by fluoroscopy can be difficult to diagnose. There are some histopathology features such as ulceration, prominent telangiectasia, and atypical stellate fibroblasts. Absence of lymphocytic infiltrate, inflammation, and presence of hyperkeratosis are helpful diagnosing this entity from others such as morphea and lichen sclerosus [5].

## 2. Case Presentation

An obese 46-year-old man with hypertension, dyslipidemia, and severe coronary artery disease referred a history of fluoroscopically guided stent implantation angioplasty six months before his Dermatology consultation. His medications included nebivolol, cilostazol, clopidogrel, and rosuvastatin. He referred an erythematous patch over his left scapula when discharged from the hospital.

**Figure 1:**
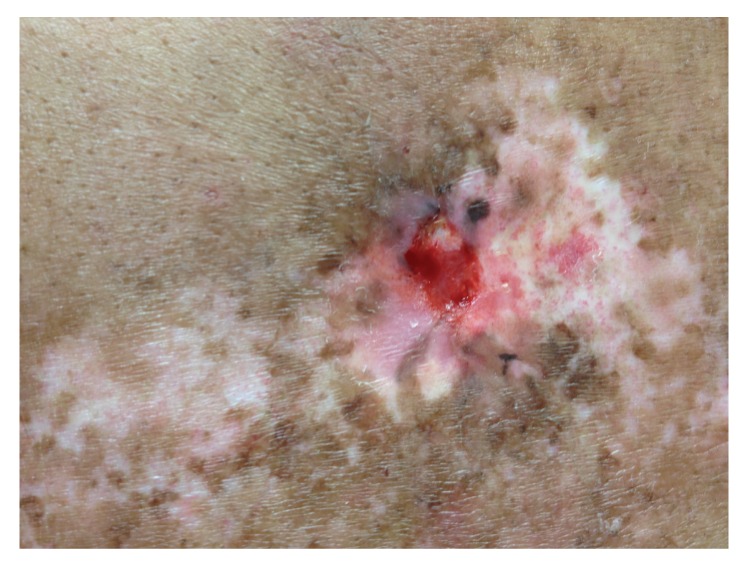
Ulcered, atrophic plaque with hypopigmentation and hyperpigmentation, as well as superficial telangiectasia.

**Figure 2:**
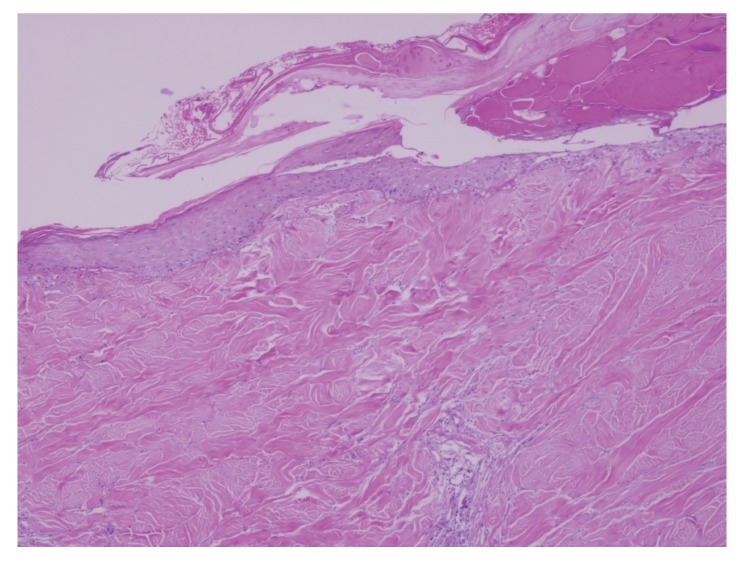
Hematoxylin and eosin. 5X. Atrophic epidermis with necrosis and central ulceration. Dermal sclerosis, loss of dermal appendages.

**Figure 3:**
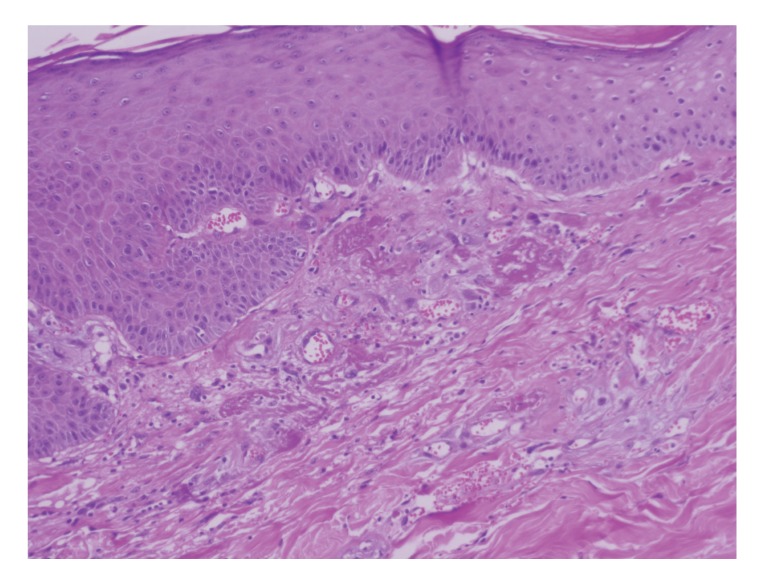
Hematoxylin and eosin, 10X. Epidermis with acanthosis and superficial prominent telangiectasia, with fibrin and fibrosis.

**Figure 4:**
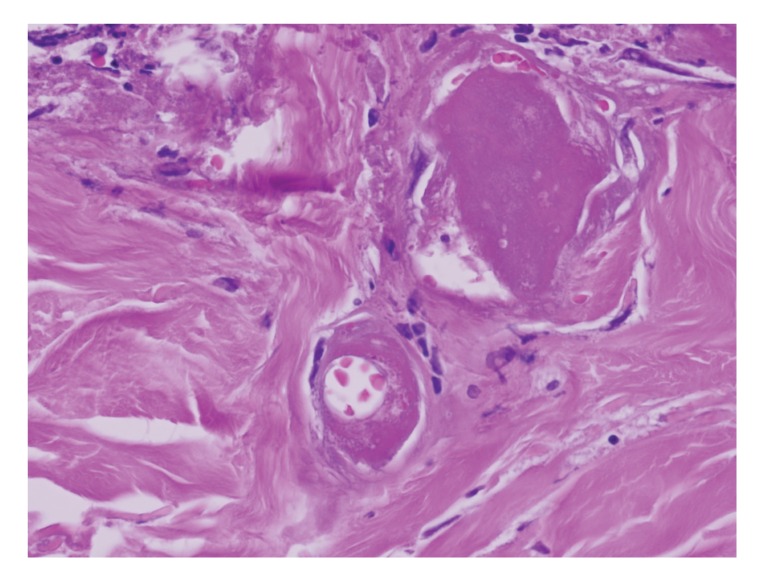
Hematoxylin and eosin. 40X. Superficial dermal telangiectasia with fibrin thrombi and fibrosis.

The lesion evolved in 3 months into an atrophic plaque that was pruritic, tender, and painful. Over the following 3 months, the lesion became indurated, ulcerated, developing hypopigmentation and hyperpigmentation, as well as superficial telangiectasia. The lesion was well demarcated, 8 × 5 cms (Figure 1). A skin biopsy specimen demonstrated changes consistent with chronic radiation dermatitis (Figures 2, 3, and 4). The histological findings, along with the location over the left scapula and the history of fluoroscopic exposure during cardiac catheterization, led to the clinical diagnosis of fluoroscopy-induced chronic radiation dermatitis.

The ulcer was treated with bismuth subgallate powder, applied every four days and left under occlusion. The ulcer resolved after thirty days of treatment. A hydrophilic ointment was indicated on the rest of the plaque.

## 3. Discussion

This case emphasizes the importance of fluoroscopic procedures as a cause of radiation dermatitis. The diagnosis of fluoroscopy-induced chronic radiation dermatitis should be considered in patients with a recent vascular lesion or morphea-like lesion, or an unexplained ulcer localized over previously radiated sites [6].

Follow-up is important after procedures that include radiation exposure [4]. Radiodermatitis has been described during many other vascular procedures like radiofrequency catheter ablation, renal angioplasty, interventional neuroradiology, implantation of cardiac resynchronization devices, and implantable cardioverter defibrillator pacemaker systems.

Radiation dermatitis' treatment outcome is limited. Simple skin grafting often fails because of poor vascularity.

With the increase of minimally invasive procedures involving fluoroscopy, radiation dermatitis is becoming more prevalent. Though rare, radiation dermatitis must always be considered as a complication of fluoroscopic procedures. Physicians involved in this type of interventions should be aware of side effects and implement measures to minimize exposure time in order to prevent development of radiation skin injuries [7].
